# Lost productivity associated with headache and depression: a quality improvement project identifying a patient population at risk

**DOI:** 10.1186/s10194-020-01107-4

**Published:** 2020-05-11

**Authors:** Virginia B. Baker, Christopher B. Sowers, Nawaz K. Hack

**Affiliations:** 1 Walter Reed National Military Medical Center, Neurology Department, 8901 Rockville Pike, Bethesda, MD 20889 USA; 2 Naval Medical Center San Diego, Anesthesiology Department, San Diego, CA USA

**Keywords:** Migraine, PHQ-9, Multi-disciplinary, Productivity, Mild traumatic brain injury (mTBI)

## Abstract

**Objective:**

This quality improvement project was implemented in order to highlight the association between headache, mTBI and depression on lost productivity and resource utilization.

**Background:**

Mood disorders, environment and traumatic brain injury are common in patients with headache, and have been shown to influence clinical course, treatment response and outcome. Although widely recognized, the association of these factors on clinical outcomes, resource utilization and productivity is not well understood.

**Methods:**

All patients presenting to a military referral center for migraines are assessed for presence of traumatic brain injury, Headache Impact Score (HIT-6) and Patient Depression Questionnaire (PHQ-9). Based on screening, patients are offered referral to mental health and a multidisciplinary headache education course.

**Results:**

237 patients were seen for headache or migraine. 180 patients had severely disabling headaches. These patients accounted for 146 emergency room visits over the course of one year. Of headache patients, 65% met criteria for depression and 15% of patients had severe depression. Only 37% of these patients carried a formal diagnosis of depression and 38% had been seen by mental health. Lost productivity and duty limitations were significantly associated with severity of depression. In service members screening positively for mild, moderate or severe depression, duty restrictions had been placed on 8.3%, 32.5% and 53.8%, respectively. Only 3.8% of patients who did not screen for depression had similar duty limitations. A history of mTBI strongly correlated with comorbid depression. Lost productivity and duty limitations were not impacted by other headache characteristics or HIT-6 scores.

**Conclusions:**

This quality improvement project identified a practice gap for treatment of comorbid depression in patients presenting to Neurology for headache. Depression strongly correlated with productivity loss, highlighting a possible target for the economic burden of headache.

## Background

Migraines and headaches affect one in seven Americans annually [1, 2]. In 2010, headache accounted for greater than 3% of all emergency room visits, and was documented as the fourth leading cause of visits to the emergency department within the United States [1]. This has considerable financial implications, through utilization of healthcare resources and lost productivity in the workplace [3, 4]. Indirect costs, including disability, lost productivity and absence from work account for more than 50–70% of the economic burden from headaches [5–7]. Within the military, headache accounts for greater than 50% of outpatient Neurology encounters. It is a cause for medical attention in 4% of all service members, placing significant demands on the healthcare system [8].

Mood disorders are common in the headache population, and have been shown to influence clinical course, treatment response and outcome [9–12]. Patients with migraine are 2–4 times more likely to develop major depressive disorder [13, 14]. It is theorized that environmental stressors, work demands, changing sleep cycles and the frequency of traumatic brain injury magnify the burden of headaches and psychiatric conditions [15–18]. Furthermore, major depressive disorders and pain syndromes have biological associations with levels of thyroid hormones, prolactin and serotonin. These markers may also be associated with risk for suicide [19]. Suicide has far-reaching psychological implications for not only the patient, but also his or her successors, leading to significant morbidity, mortality and use of healthcare resources [20]. Despite this recognized association and grave impact, the relationship between headaches and depression is not well understood [21–25].

Concurrent treatment of both headache and depression is supported through growing research and literature [26, 27]. Our Quality Improvement project initiated at Walter Reed National Military Medical Center (WRNMMC) instituted a screening tool to better understand the relationship between headache, psychiatric comorbidities and productivity. This was further stratified by the presence of mild traumatic brain injury (mTBI). Neurology promoted a multidisciplinary approach to care through group education and interdisciplinary involvement [24, 28]. This strategy was implemented to decrease resource utilization and loss of productivity among service members and their dependents. This is the first paper to investigate the interplay between migraines, mTBI and depression on lost productivity in the United States [29, 30].

## Methods

From August 2018 until June 2019, an IRB-approved Quality Improvement Project was initiated at Walter Reed National Military Medical Center (WRNMMC) in order to screen for and treat co-morbidities in patients presenting for headache. All patients presenting to the Neurology clinic for a diagnosis of headache or migraine were administered a headache intake questionnaire. Exclusion criteria included patients with a diagnosis of a secondary headache disorder or a history of moderate or severe traumatic brain injury. Only patients who are active duty, active duty dependents or military retirees are eligible for care at WRNMMC.

Headache intake questionnaires included the Headache Impact Test (HIT-6) and Patient Health Questionnaire (PHQ-9). HIT-6 score is a validated tool for headache, including migraine and chronic migraine. Scores range from 36 to 78 and correlate with the level of impact on daily life and ability to function. Scores greater than or equal to 60 indicate a severe impact on patient life [31, 32]. The PHQ-9 is a validated, self-administered scoring system used to screen for depression. Scores range from 0 to 29, with a score greater or equal than 5, 10, 15, and 20 representing mild, moderate, moderately severe and severe depression, respectively [33, 34]. Scores greater than or equal to 10 have been shown to have a sensitivity of .88 and specificity of .85 for a diagnosis of Major Depressive Disorder [35]. In addition, we surveyed utilization of healthcare resources through hospital admissions and emergency room visits. Productivity was assessed through documentation of duty restrictions, limited duty status or medical separation from the military due to headache or migraine. Financial implications were estimated based on a computational analysis by the National Defense Research Institute (RAND) published in 2007. This analysis calculated individual costs based on active duty pay, benefits and retiree compensation [36].

Patients were evaluated for medical and psychiatric comorbidities as well as utilization of healthcare resources using intake forms and physician interview. Based on this information, referral to mental health was offered and a migraine education class was encouraged. The migraine education class emphasized a multidisciplinary approach to care, utilizing mental health, psychiatry, physical therapy and sleep therapy counterparts. It stressed the importance of lifestyle modifications in migraine prevention and management, to include stress reduction, trigger avoidance and implementation of healthy routines including scheduled hydration, exercise and sleep. Referrals to psychiatry were also encouraged for all patients who screened positively for depression. However, the specifics of treatment and management of these psychiatric comorbidities were not reviewed. This project was implemented to increase recognition and improve access to care for comorbid psychiatric conditions in headache patients. Ultimately, we hoped to highlight that the prevalence of depression and insufficient access to mental health services had a negative impact on resource utilization.

## Results

From August 2018 until June 2019, 237 patients were seen in a Neurology tertiary care center for headache or migraine and 208 completed depression screening. Of these patients, 77% were active duty or retired military personnel. No patients evaluated had a history of moderate or severe traumatic brain injury or secondary headache disorder. 32% had a history of a prior mild traumatic brain injury (mTBI). The population averaged 16 headache days per month. Average headache duration was 15 h (Table 1). Headache impact score averaged 64 points, indicating a severe impact on patient life. 180 patients had severely disabling headaches. Of the 174 active duty personnel, 36 were undergoing a medical board for headaches, meaning that they were no longer qualified to perform as required for military duty. Over the course of one year, this population accounted for 146 emergency room visits for acute treatment of headache or migraine.

**Table 1:** Patient demographics. A comparison of duty status, headache diagnosis, TBI history and medication use in patients who screened positively versus negatively for depression

Characteristic	No Depression (73)	Depression (135)
Duty Status – no. (%)		
Active	38 (52%)	67 (50%)
Retired	21 (29%)	34 (25%)
Dependent	14 (19%)	34 (25%)
Diagnosis – no. (%)		
PTHA	8 (11%)	40 (30%)
Total Migraine	65 (89%)	95 (70%)
Episodic Migraine	39 (53.4%)	33 (24%)
Chronic Migraine	2 (35.6%)	62 (46%)
Mild TBI – no. (%)		
History mTBI	11 (15%)	55 (41%)
Headache Characteristics		
Frequency – day / mo.	12	18
Duration – hrs	10.2	17.7
Medication Use – no. (%)		
BOTOX	32 (44%)	73 (54%)
Taking Oral Preventatives	36 (49%)	80 (59%)
Taking Oral Abortives	69 (95%)	15 (89%)

**Fig. 1:**
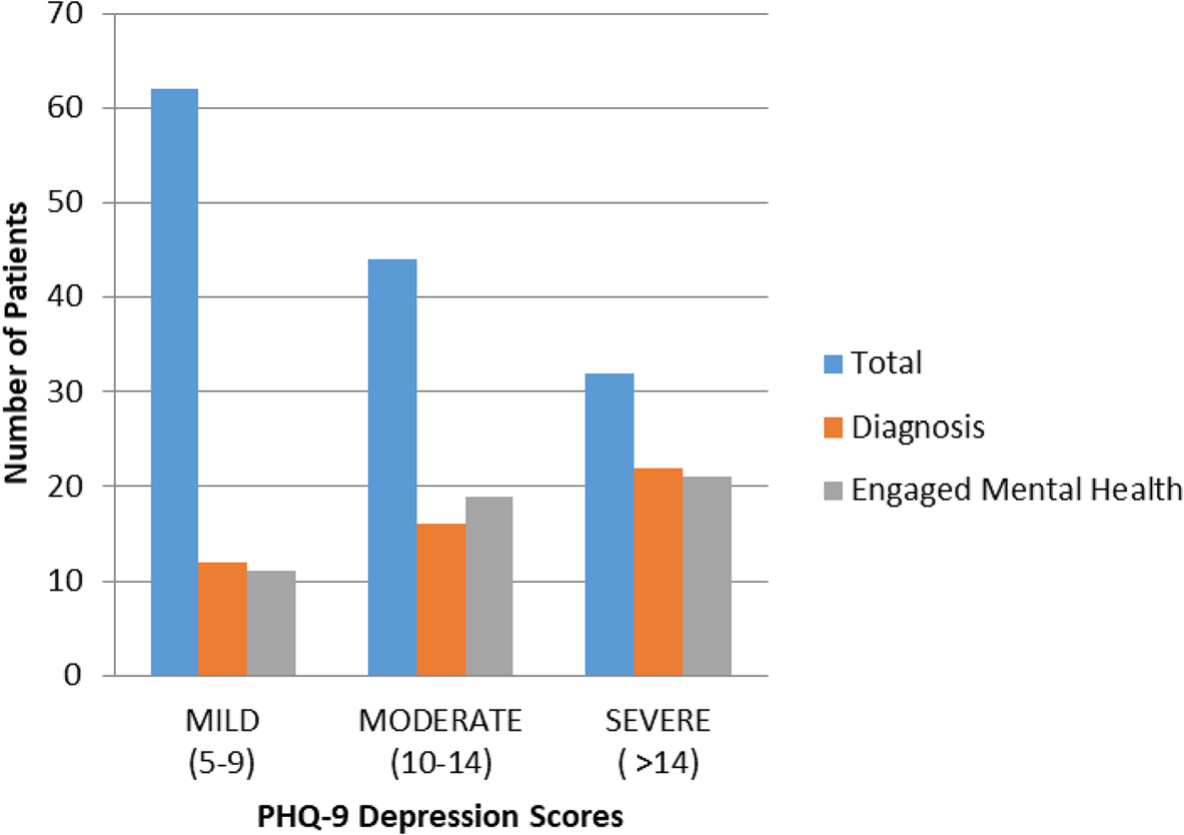
Practice gap in patients screening positively for depression. Patients who screened positively on PHQ-9 for depression were evaluated for a formal diagnosis made by a medical provider and for engagement of mental health services

In the headache population, average PHQ-9 self-assessment score was 8, consistent with a diagnosis of mild depression. PHQ-9 scores were positive for depression in 65% of screened patients presenting for a primary headache disorder. Of patients who met criteria, 37% had a formal diagnosis of depression made by a medical provider and 38% had been engaged with mental health at the time of presentation to Neurology (Fig. 1). 15% of patients met criteria for severe depression. Of patients meeting criteria for severe depression, 71% had a documented diagnosis and 68% of these patients were engaged with mental health. Depression was more likely in patients who had a history of prior mTBI, with a prevalence of 81%. Of patients with a history of mTBI and depression, 49% had a diagnosis and 45% were engaged with mental health services. Post-traumatic stress disorder was documented in 14% of this patient population.

Patients were stratified based on the presence and severity of depression, and statistical analysis was performed using chi-squared and ANOVA tests. There was no significant difference in headache characteristics or likelihood to present to the emergency room for headache treatment. Headache impact test (HIT-6) averaged 61 in patients without depression. Patients with mild, moderate and severe depression had a HIT-6 score of 63, 67 and 67 respectively. There was positive trend in HIT-6 score based on depression severity, but this did not meet statistical significance (supplemental figure 1). History of mTBI did not impact headache days, headache duration or HIT-6 score, but did correlate with higher depression rates.

**Fig. 2:**
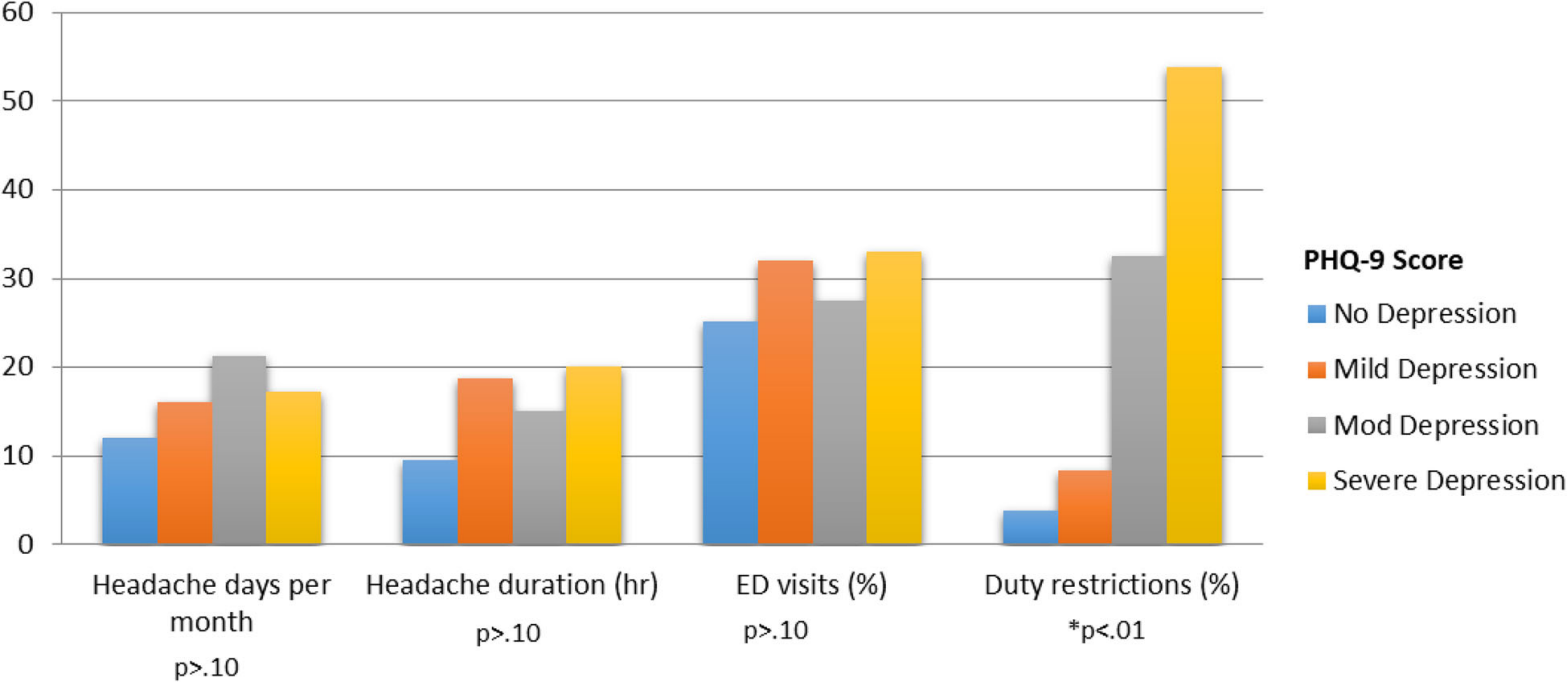
Headache characteristics based on depression severity. Headache characteristics and duty restrictions were stratified based on the presence and severity of depression, as detected by PHQ-9 scores. No headache characteristics had statistically significant correlation with depression severity. Only duty restrictions were significantly associated with severity of depression based on PHQ-9

Patients with higher depression scores were significantly more likely to have active duty work restrictions or be medically boarded for migraines. Duty restrictions placed as a result of headache or migraine occurred in 3.8% of patients who did not meet criteria for depression. In patients with depression, 29% had similar duty restrictions. In patients scoring for mild, moderate or severe depression, duty restrictions had been placed on 8.3%, 32.5% and 53.8%, respectively (Fig. 2). Patients with higher severity of depression had a significant increase in the number of medical boards and duty limitations as a result of headache. Of all patients undergoing a medical board or limited duty restrictions for headaches, 94% had a PHQ-9 score consistent with a diagnosis of depression. There was no change in the number of medical boards or duty limitations based on HIT-6 scoring system or other headache characteristics including headache days or duration.

## Discussion

Twenty percent of our active duty cohort was facing duty restrictions with potential for medical separation. Based on RAND and Secretary of Defense estimates, an enlisted service member’s military separation accounts for a lifecycle cost of greater than $860,000 and an officer member’s costs account for greater than $1,700,000 [36]. Medical boards within our cohort accounted for at least 31 million dollars of resource utilization and lost productivity. Of service members who screened positive for depression, nearly 30% were facing duty restrictions or medical separation from the military. Interestingly, of members who did not screen positive for depression, only 3.8% had similar duty restrictions. The military provides a unique patient population for early detection of lost productivity, given required reporting of medically-related duty limitations. This provides a quantifiable metric for lost productivity and career limitations and identifies possible improvements for clinical practice. Our study identifies comorbid depression as a possible target for the lost productivity, career impact and substantial financial implications as a result of headache.

Although it is recognized that headaches and migraines are a major cause for lost productivity and increased utilization of resources, this has not been evaluated concurrently with depression [37, 38]. In this patient population, 65% screened positive for depression and 15% met criteria for severe depression. Military duty restrictions and lost productivity correlate greater with depression than with headache impact scores, despite patients presenting for a primary complaint of headache. A limitation of this manuscript includes the lack of information regarding comorbid anxiety, which is also known to have high correlation with migraine and headaches. In future research, we recommend also utilizing a screening tool for anxiety, such as the Generalized Anxiety Disorder (GAD-7). Presence of post-traumatic stress disorder in 14% of our patient population may indicate that comorbid anxiety and arousal conditions were coexisting.

Less than half of patients who scored positive on PHQ-9 had a mental health diagnosis and even fewer were engaged with mental health services. Recognition and treatment were more likely if patients met criteria for severe depression. Even in this subgroup, nearly one-third were without a formal diagnosis or engaged with mental health. This practice gap presents an opportunity for Neurologists to improve patient safety, quality of life and productivity. We propose that all patients presenting to Neurology for treatment of headache or migraine be screened for psychiatric comorbidities. Patients who screen positively should be referred to behavioral health or psychiatry for early evaluation and treatment. Our screening system promotes a multimodal approach to care which improved access to mental health services in patients presenting for headaches. We believe that early recognition and treatment of depression may improve productivity and maintain deployability of our armed forces and their dependents [39].

A limitation of this data is that the results may not be generalizable to the United States population as a whole. Our population had been exposed to unique environmental and physical factors, including changing environments, inconsistent schedules, atypical work conditions and higher frequency of traumatic brain injuries. These factors have been shown to affect headaches and migraines, and may predispose patients to higher PHQ-9 or HIT-6 scores. In our subgroup analysis, traumatic brain injury was independently associated with both depression and lost productivity. One-third of patients presenting for headache or migraine had a diagnosis of prior mild traumatic brain injury. Patients with a history of traumatic brain injury were more likely to meet criteria for depression, which may contribute to the loss of productivity or limitations of duty in this population. The association of traumatic brain injury with heightened PHQ-9 scores highlights the impact of military-specific stressors on patient outcomes.

This Quality Improvement project can serve as a proof of concept for future research and a possible target for growing healthcare costs. This small cohort accounted for a revenue loss of 31 million dollars. The estimated cost to the United States healthcare system would be in the billions [6]. Treatment of comorbid psychiatric conditions may have considerable financial implications in the headache population, especially within the United States military population. The study utilized a standardized and validated screening tool to detect comorbid psychiatric conditions in patients presenting for headache. Through recognition of comorbid depression, access to care for mental health services was improved. Further research is needed to define the effect of treating depression on productivity and healthcare costs in patients with headache or migraine.

## Conclusions

This study is the first to present the relationship between migraine, depression and traumatic brain injury on lost productivity. We propose a concept model for all Neurology clinics, which utilizes screening tools as a basis for mental health referrals and involvement in a multidisciplinary headache education course. In the headache population, depression appears to have a high predictive value for productivity loss. Early treatment and detection can improve quality of life, enhance productivity and decrease the use of medical resources.

## Supplementary information

**Additional file 1: Supplemental figure 1.** Correlation of HIT-6 score with severity of depression. Although not statistically significant, Headache Impact Test (HIT-6) had a correlation with the severity of depression.

## Data Availability

Available under request from the corresponding author.

## Abbreviations

HIT-6: Headache Impact Test
PHQ-9: Public Health Questionnaire
TBI: Traumatic brain injury
mTBI: mild traumatic brain injury
WRNMMC: Walter Reed National Military Medical Center
