# Supplementary material for: Lost productivity associated with headache and depression: a quality improvement project identifying a patient population at risk
Source: J Headache Pain. 2020 May 11;21(1):50. doi: 10.1186/s10194-020-01107-4 (PMC7216618; doi:10.1186/s10194-020-01107-4)

Supplemental figure 1: Correlation of HIT-6 score with severity of depression.

Although not statistically significant, Headache Impact Test (HIT-6) had a correlation with the severity of depression.


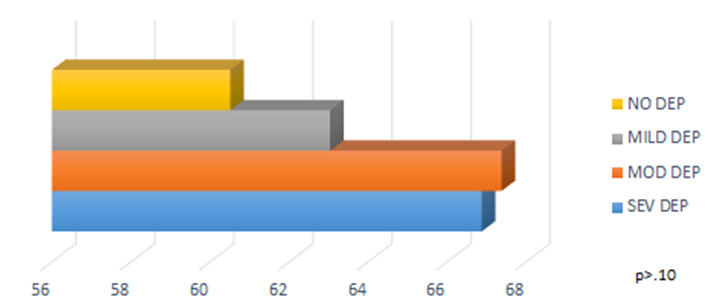

Supplement: Supplementary file 1 — Additional file 1: Supplemental figure 1. Correlation of HIT-6 score with severity of depression. Although not statistically significant, Headache Impact Test (HIT-6) had a correlation with the severity of depression. [file 10194_2020_1107_MOESM1_ESM.docx]
